# Small cell carcinoma of the anus: a case report

**DOI:** 10.1186/1757-1626-2-9396

**Published:** 2009-12-24

**Authors:** Sudeendra Doddi, Tarun Singhal, Collette De Silva, Frank Smedley, Prakash Sinha, Martin Leslie

**Affiliations:** 1 Department of Surgery, Princess Royal University Hospital, Orpington, Greater London, BR6 8ND, UK; 2 Department of Clinical Oncology, Princess Royal University Hospital, Orpington, Greater London, BR6 8ND, UK

## Abstract

Small cell carcinoma of the anus is a very rare but aggressive tumour. We present a case of a 60-year old lady with small cell carcinoma of the anus. She had no metastatic disease on presentation. She had chemotherapy and radiotherapy but developed distant metastasis after completion of treatment. Immunohistochemistry is required to make a diagnosis. Chemotherapy remains the mainstay of treatment for small cell carcinoma of the anus with or without metastatic disease. Radiotherapy is for local control and relief of symptoms.

## Introduction

Anal cancer is a rare tumour of the gastrointestinal tract representing only about 2% of the anorectal malignancies [1]. Most common anal cancers are squamous cell carcinomas and adenocarcinomas. Less common ones are basaloid carcinoma, melanoma, leiomyosarcoma and small cell carcinoma [1]. The latter, though very rare, is clinically important because of its aggressive clinical course with a tendency for early distant metastases. Small cell carcinomas of the anus are oncologically similar to their counterparts in the lungs and are therefore treated along the same lines.

## Case presentation

**Figure 1:**
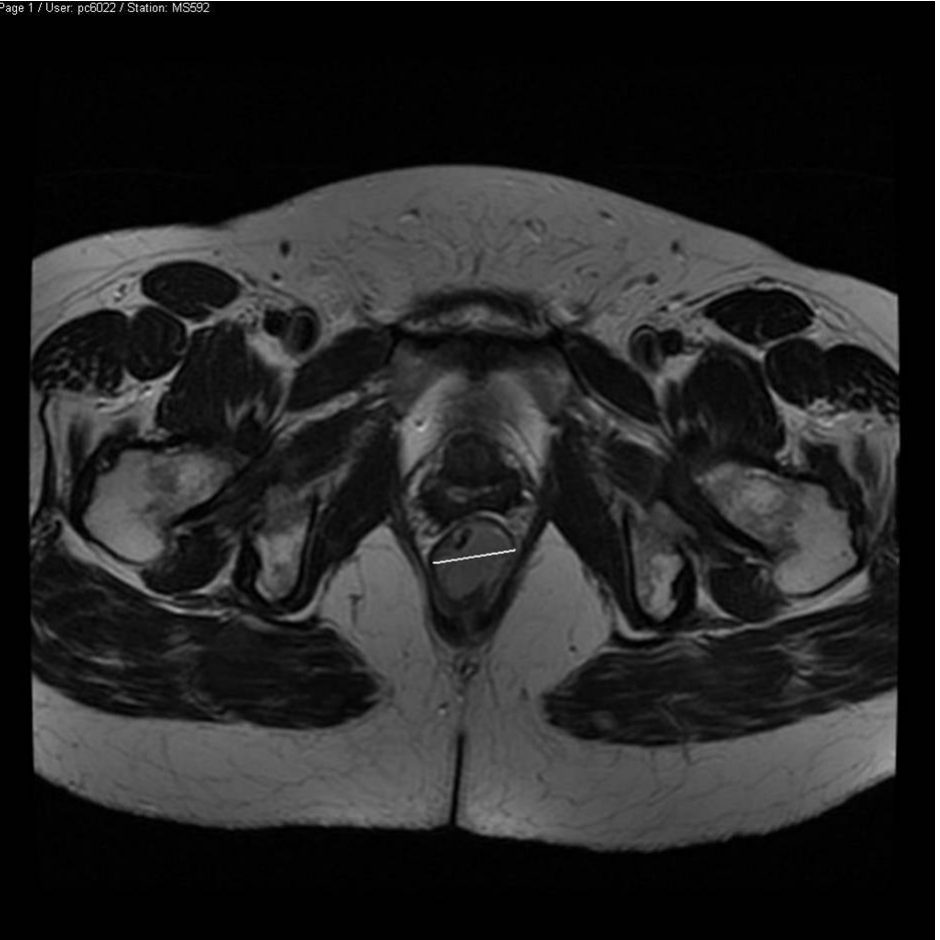
**MRI of the anal canal showing the tumour**.

**Figure 2:**
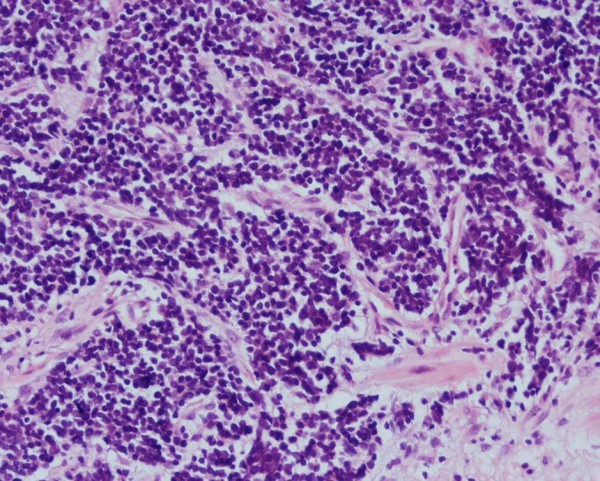
**Haematoxylin and eosin staining of the small cell carcinoma of the anus**.

**Figure 3:**
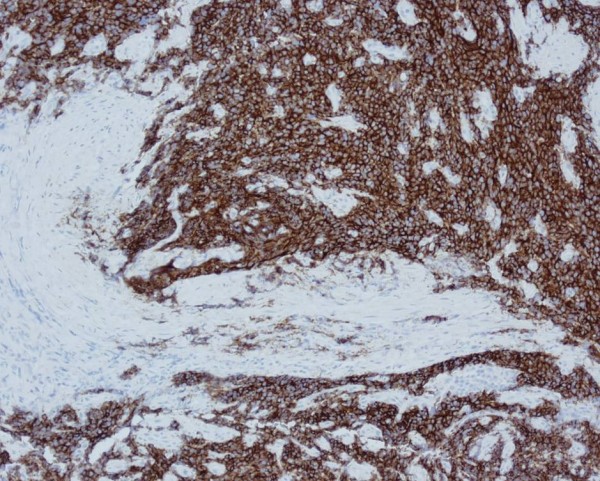
**Immunohistochemical staining of the small cell carcinoma of the anus**.

A 60-year old African lady originally from Ghana presented with a short history of rectal bleeding and anal pain. Over the past three months, she had noticed tenesmus and increased frequency of bowel movements, as well as anorexia and weight loss. There was no significant past or family history. She was a non-smoker. On rectal examination there was a palpable mass which bled on contact. There were no clinical features of bowel obstruction. An MRI of the anus showed a 2.7 cm tumour in the posterior aspect of the distal anal canal (Figure 1). At examination under anaesthetic (EUA) the mass in the anus was confirmed and biopsies were taken. The histopathology showed small malignant cells with hyperchromatic nuclei and scanty cytoplasm on haematoxylin and eosin staining (Figure 2). On immunostaining, the malignant cells were strongly positive for CD56 (Figure 3) and showed weak focal positivity for CAM 5.2 and MNF. There was negative staining for CK7, CK20, thyroid transcription factor 1 (TTF-1) and leukocyte common antigen. These results were consistent with a primary small cell carcinoma of the anus. The patient was not tested for HIV and tumour markers were not measured. Staging CT and PET scans showed no evidence of metastatic disease. The patient underwent treatment with six cycles of chemotherapy using cisplatinum and etoposide followed by a course of radical radiotherapy to the anus and pelvis. Following treatment there was thickening at the primary tumour site; however two EUA's and biopsies did not show any residual tumour. Twelve months after completion of treatment the patient relapsed with liver and lung metastasis although the primary site remained clear. No response was seen to palliative chemotherapy and the patient succumbed to metastatic disease 18 months following the initial diagnosis.

## Discussion

Small-cell carcinoma of the lungs, first described in 1926, accounts for about 20% of all lung cancers whereas extra-pulmonary small cell carcinomas are uncommon, with an incidence of approximately 0.4% [2]. Approximately 544 cases of gastro-intestinal small cell carcinomas were reported in the English literature till 2004 [3]. The oesophagus is the most common primary site followed by colorectal [4].

Less than 1% of anal cancers are small cell carcinomas-the most common being squamous cell carcinomas (74%) and 19% are adenocarcinomas [5].

These tumours have been documented in HIV positive patients, and those with a history of exposure to radiation [6]. Neuroendocrine (NE) tumours can be found incidentally within adenomatous polyps [7,8]. To diagnose small cell carcinoma, positive immuno-reactivity to neuroendocrine markers is required-the most reliable being synaptophysin [9]. Other markers include CD56 and Leu-7/CD57. CD56 is a neural cell adhesion molecule that is positive in small cell carcinomas of different sites including the lung and gastrointestinal tract. The use of thyroid transcription factor (TTF)-1 will help separate a primary anal canal small cell carcinoma from a lung metastasis, as it is positive in the latter. Systemic symptoms are common; ectopic hormonal secretion may occur.

These tumours are highly aggressive, with lymph node, liver and lung metastasis at presentation even when the primary tumour is limited to the submucosa or mucosa [10]. Metastatic disease was detected at the time of diagnosis in 69% percent of the patients in the study by Bernick et al [9]. The 6-month survival is 58% and 5-year survival is 6% [11]. In contrast, for squamous cell carcinoma of the anus, the overall survival rate for advanced tumours is 50-60% at 5 years [12]. In anal small cell carcinoma with or without metastatic disease at presentation, chemotherapy is the main modality of treatment. It is Cisplatinum-based and usually includes etoposide, cyclophosphamide and doxorubicin. Radiotherapy is mainly for local control and relief of symptoms [1]. The response to chemotherapy is 70% to 90% though transient. Typically, a good response to initial therapy is shortly followed by relapse or rapid progression with a median survival of only 6-12 months [1]. Though our patient did not present with distant metastasis, she developed lung and liver secondaries a year after completion of treatment. At this stage there was no local recurrence. Her clinical course matched what has been described in previous case reports.

## Conclusion

Small cell carcinoma of the anus is very rare. Immunocytochemistry is needed for diagnosis. Chemotherapy is the mainstay of treatment even though the response is short lived. Radiotherapy is mainly for local control and relief of symptoms. The prognosis is poor with frequent early distant metastasis despite local control.

## Consent

Written informed consent was obtained from the patient's next of kin for publication of this case report and accompanying images. A copy of the written consent is available for review by the Editor-in-Chief of this journal.

## Competing interests

The authors declare that they have no competing interests.

## Authors' contributions

SD, TS, CD and FS were involved in obtaining patient details and images, literature search and drafting the manuscript. PS and ML were involved in writing the manuscript. All authors read and approved the final manuscript.

## References

[B1] Meyer A Bruns F Richter K Grünwald V Karstens JH Small cell cancer of the anal canal-case report of a rare tumour Anticancer Res 2007 2 1047 1050 17465242

[B2] Cicin I Karagol H Uzunoglu S Uygun K Usta U Kocak Z Caloglu M Saynak M Tokatli F Uzal C Extra pulmonary small-cell carcinoma compared with small-cell lung carcinoma: a retrospective single-center study Cancer 2007 110 5 1068 1076 10.1002/cncr.22887 17614337

[B3] Brenner B Tang LH Klimstra DS Kelsen DP Small-cell carcinomas of the gastrointestinal tract: a review J Clin Oncol 2004 22 13 2730 2739 10.1200/JCO.2004.09.075 15226341

[B4] Huncharek M Muscat J Small cell carcinoma of the oesophagus. The Massachusetts General Hospital experience Chest 1995 107 179 181 10.1378/chest.107.1.179 7813272

[B5] Beahrs OH Wilson SM Carcinoma of the anus Ann Surg 1976 184 4 422 428 10.1097/00000658-197610000-00004 189707 PMC1345433

[B6] Nakahara H Moriya Y Shinkai T Hirota T Small cell carcinoma of the anus in a human HIV carrier: report of a case Surg Today 1993 23 1 85 88 10.1007/BF00309007 8384908

[B7] Ihtiyar E Algin C Isiksoy S Ates E Small cell carcinoma of rectum: a case report World J Gastroenterol 2005 11 20 3156 3158 15918209 10.3748/wjg.v11.i20.3156 PMC4305859

[B8] Izuishi K Arai T Ochiai A Ono M Sugito M Tajiri H Saito N Long-term survival in advanced small cell carcinoma of the colorectum: report of a case Surg Today 2002 32 1 72 74 10.1007/s595-002-8118-8 11871823

[B9] Bernick PE Klimstra DS Shia J Minsky B Saltz L Shi W Thaler H Guillem J Paty P Cohen AM Wong WD Neuroendocrine carcinomas of the colon and rectum Dis Colon Rectum 2004 47 2 163 169 10.1007/s10350-003-0038-1 15043285

[B10] Balachandra B Marcus V Jass JR Poorly differentiated tumours of the anal canal: a diagnostic strategy for the surgical pathologist Histopathology 2007 50 1 163 174 10.1111/j.1365-2559.2006.02550.x 17204029

[B11] Saclarides TJ Szeluga D Staren ED Neuroendocrine cancers of the colon and rectum. Results of a ten-year experience Dis Colon Rectum 1994 37 7 635 642 10.1007/BF02054405 8026228

[B12] Glynne-Jones R Mawdsley S Anal cancer: is neoadjuvant cisplatin chemotherapy or chemoradiotherapy friend or foe? Nat Clin Pract Oncol 2008 5 12 692 693 10.1038/ncponc1258 18852720

